# Prevalence and correlates of depression, anxiety, and stress in medical residents of a Brazilian academic health system

**DOI:** 10.1186/s12909-019-1621-z

**Published:** 2019-06-11

**Authors:** Paula Lage Pasqualucci, Luciana Luccas Mendes Damaso, Arthur Hirschfeld Danila, Daniel Fatori, Francisco Lotufo Neto, Vera Hermina Kalika Koch

**Affiliations:** 1 Instituto da Criança, Universidade de São Paulo, São Paulo, Brazil; 2 Instituto de Psiquiatria, Universidade de Sao Paulo, 1967, Oscar Freire, São Paulo, 05409011 Brazil; 3 Comissao de Residencia Medica (COREME), Universidade de Sao Paulo, São Paulo, Brazil

**Keywords:** Medical residents, Mental health, Burnout syndrome, Quality of life

## Abstract

**Background:**

Several studies correlate medical residency with the occurrence of mental health disorders, Burnout Syndrome and quality of life impairment. It has been demonstrated that mental health disorders increase medical errors and lead to less effective patient care. Considering such context, this study aimed to evaluate the prevalence of anxiety, depression, stress and to identify its correlates with Burnout Syndrome and quality of life in a sample of medical residents and fellow physicians of the largest Brazilian academic health system.

**Methods:**

In 2017, 1648 participants were voluntarily and anonymously surveyed online about demographic characteristics, Burnout Syndrome, mental symptoms, and quality of life measured by validated questionnaires. Responses were captured through REDCap platform and multivariate statistical analyses were performed with STATA 15.

**Results:**

A total of 606 (36.8%) residents/fellows physicians completed the survey. Depression symptoms were present in 19%, anxiety symptoms in 16% and stress symptoms in 17.7% of the sample. Burnout Syndrome was present in 63% of the sample. Multivariate analysis showed a statistical significant positive correlation between Burnout Syndrome and depression, anxiety and stress symptoms and a negative correlation between mental symptoms and quality of life scores.

**Conclusions:**

Mental health symptoms prevalence in this study is similar to other studies and their occurrence is positively correlated with Burnout Syndrome among medical residents/fellow physicians of the largest Brazilian academic health system. These results are relevant and must be confirmed by multicentric longitudinal studies. This study reinforces the importance of debating interventions to improve mental health among doctors in training.

## Background

Recent studies correlate medical residency with the development of mental health disorders, occurrence of Burnout Syndrome and quality of life impairment among medical residents/fellows [1–5]. It has been demonstrated that mental health and quality of life impairments increase medical errors and lead to less effective patient care [6–10].

In Brazil, the existing scientific literature in the field confirms high levels of Burnout Syndrome and mental health and quality of life impairments among Brazilian medical residents/fellows of different parts of Brazil and of diverse clinical settings [11–14].

However, existing studies in Brazil are mostly limited because of small sample sizes and undiversified study population from a single specialty field or year of training.

Therefore, new studies with larger and more diversified samples are necessary to improve knowledge of Brazilian medical resident’s/fellow’s mental health status. The aim of this study is to perform a cross-sectional study to evaluate the prevalence of anxiety, depression and stress symptoms in a diverse sample of medical residents/fellow physicians of different specialty fields and training years from the largest Brazilian academic health system and try to identify its correlates with Burnout Syndrome and quality of life.

## Method

### Participants

From September to November of 2017, a total of 1648 medical residents and fellow physicians of all specialty fields of Faculty of Medicine, University of São Paulo, Brazil, received an invitation by e-mail to participate in our study, along with a link to an online survey via Research electronic data capture (REDCap) [15]. We sent the same e-mail up to three times during the study period to stimulate adherence to the survey. Participation was voluntary and all responses were anonymous. The Institutional Review Board approved the study.

### Study measures

The survey included questions about sociodemographic information (age, relationship status, place of medical graduation, specialty, year in training) and validated scales to assess Burnout Syndrome, depression, anxiety and stress symptoms and quality of life.

The medical specialties were categorized in three groups: surgery specialties (General Surgery, Surgery Subspecialties, Orthopedics, Neurological Surgery, Otolaryngology, Obstetrics and Gynecology, Ophthalmology), non clinical non surgical (Pathology, Radiology, Anesthesiology, Acupuncture, Legal Medicine, Nuclear Medicine), clinical specialties (all specialties not included in the other two categories).

### Burnout syndrome

In order to verify the existence of Burnout symptoms, the survey included the Maslach Burnout Inventory (MBI), which contains three subscales (emotional exhaustion, depersonalization, and sense of personal accomplishment). The subscale scores were categorized according to criteria adapted for the Brazilian population [16]: emotional exhaustion (low 0–15, medium 16–25, and high 26–54), professional performance (low 0–33, medium 34–42, high 43–48) and depersonalization (low 0–2, medium 3–8, high 9–30). Burnout Syndrome was considered to be present in subjects with high scores in emotional exhaustion subscale.

### Mental health symptoms

The Depression, Anxiety and Stress Scale-Short Form (DASS-SF) [17] was chosen as the instrument to evaluate mental health symptoms. It is a brief version of a scale developed to measure the severity of depression, anxiety and stress symptoms. This instrument was selected for its ability to discriminate between these three categories and indicate severity. The categorization of DASS-SF was carried out according to the instructions in the official scoring guide of the scale. We considered subjects who scored for severe and extreme symptoms positive for depression, anxiety and stress.

### Quality of life

The World Health Organization Quality of Life-Bref (WHOQOL-BREF) [18] was the instrument used to measure markers related to quality of life. This is the brief version of an instrument created by the World Health Organization for this purpose. The questionnaire is organized in four domains: psychical and psychological health, social relationships and environment. In each of the four domains, higher scores represent better quality of life.

### Statistical analysis

Analysis of frequencies and cross-tabulations for categorical variables and central tendency measures for continuous variables were performed. The outcomes of interest were depression, anxiety and stress symptoms (according to DASS-SF).

We conducted multivariate logistic regression analyses to identify correlates with depression, anxiety and stress symptoms. Therefore, DASS-SF was dichotomized in normal/low/mild and severe/extreme scores and MBI score was dichotomized in low/normal and high scores. We considered depression, anxiety and stress symptoms to be present in those subjects with severe/extreme scores in DASS-SF and Burnout Syndrome to be present in those with high scores in emotional exhaustion MBI’s subscale.

The following independent variables were considered: age, relationship status, medical specialty (surgery, clinical, non-clinical/non-surgery), year in training, MBI’s subscales and WHOQOL domains (physical, psychological, social relations, environment, perception of quality of life, satisfaction with health).

All models presented good adjustments according to Hosmer-Lemeshow test, McFadden’s adjusted pseudo-R2 and variance inflation factors. The level of significance used for the statistical tests was *p* < 0,05. Confidence intervals of 95% were provided for statistical parameters. All analyses were performed using STATA 15.

## Results

Among those invited to participate in the study (*n* = 1648), a total of 606 residents/fellows physicians) completed the survey, which results in a participation rate of 36.8%. 177 surveys were excluded due to missing data in most variables.

The demographic characteristics of the sample are described in Table 1. The mean age was 28 years old, the majority was single (84.5%), most subjects were specializing in the clinical field (66.8%) and were in the first three years of residency (81.8%).

**Table 1:** Demographic characteristics of resident/fellow physicians, respondents to the survey

Mean age, years	28.0 (2.6)
Relationship status	N (%)
Single	512 (84,5%)
Married	94 (15,5%)
Place of medical graduation	N (%)
City of São Paulo	267 (44,1%)
Other city in São Paulo State	107 (17,7%)
Other State	232 (38,3%)
Specialty	N (%)
Surgery	158 (26.1%)
Clinical	405 (66.8%)
Non-clinical, non-surgery	43 (7.1%)
Year in training	N (%)
First year	200 (33.0%)
Second year	137 (22.6%)
Third year	159 (26.2%)
Fourth year	97 (16.0%)
Fifth year	11 (1.8%)
Sixth year	2 (0.3%)

Table 2 depicts the prevalence of mental health symptoms and Burnout Syndrome among our sample. The percentages are related to the total number of subjects who answered all DASS-SF items (*N* = 580). According to DASS-SF results, depression symptoms were present in 19% of the sample, anxiety symptoms in 16% and stress symptoms in 17.7%. According to MBI, emotional exhaustion score was high in 63% of the sample, personal accomplishment score was low in 49.2% and depersonalization score was high in 63.5% of the sample. Therefore, Burnout Syndrome was positive in 63% of the sample, as we considered Burnout Syndrome present in those with high scores for emotional exhaustion subscale.

**Table 2:** Mental health problems and Burnout prevalence

Mental Heath problems according to DASS-21	Normal	Low	Mild	Severe	Extreme
Depression	289 (49.8%)	77 (13.3%)	104 (17.9%)	44 (7.6%)	66 (11.4%)
Anxiety	391 (67.4%)	41 (7.1%)	55 (9.5%)	31 (5.3%)	62 (10.7%)
Stress	319 (55.0%)	72 (12.4%	86 (14.8%)	54 (9.3%)	49 (8.4%)
Burnout according to MBI	Low	Normal	High		
Emotional Exhaustion	85 (14.0%)	139 (22.9%)	382 (63.0%)		
Personal accomplishment	298 (49.2%)	253 (41.7%)	55 (9.1%)		
Depersonalization	67 (11.1%)	154 (25.4%)	385 (63.5%)		

The WHOQOL-BREF survey presented the following means and standard deviations (SD) on the four domains: physical health 3.5 (0.6); psychological health 3.3 (0.7); relationships 3.2 (0.8); environment 3.4 (0.6). The quality of life perception was 1 (1.0) and the overall satisfaction with quality of life was 2.9 (1.0). For this last two items, we considered the score punctuation: needs to improve (from 1 to 2.9); regular (from 3 to 3.9); good (from 4 to 4.9) e very good [5].

Three multivariate regression models were performed with all covariates of interest as presented in Table 3. The models showed a positive correlation between Burnout Syndrome and depression (OR = 2.7, CI = 1.7–4.1, *p* value < 0.000), anxiety (OR = 2.5, CI = 1.7–3.7 *p* < 0.000) and stress symptoms (OR = 2.6, CI = 1.8–4.0, *p* value = < 0.000).

**Table 3:** Logistic regression models for identification of clinical traits associated with depression, anxiety and stress

		Depression				Anxiety				Stress		
		CI 95%				CI 95%				IC 95%		
Characteristics	OR	Inferior limit	Superior limit	*P* value	OR	Inferior limit	Superior limit	*P* value	OR	Inferior limit	Superior limit	*P* value
Age	0.9	0.8	1.1	0.310	0.9	0.8	1.1	0.345	1.0	0.8	1.1	0.572
Relationship status (ref.: single)	0.9	0.4	2.1	0.781	1.4	0.6	3.0	0.408	0.8	0.3	2.0	0.638
Specialty (ref.: surgery)												
Non-clinical, non surgical	1.3	0.3	5.3	0.695	3.9	1.1	13.8	**0.034**	1.5	0.4	5.6	0.533
Clinical	0.9	0.4	2.0	0.864	1.8	0.9	3.7	0.127	1.1	0.5	2.3	0.836
Place of graduation (ref.: São Paulo)												
Other city in SP State	0.9	0.3	2.2	0.761	1.1	0.5	2.5	0.750	1.3	0.6	3.0	0.548
Other States	1.3	0.6	2.6	0.502	0.6	0.3	1.2	0.166	0.6	0.3	1.2	0.152
Year in training (ref.: first year)												
Second	0.6	0.3	1.5	0.296	0.7	0.3	1.4	0.277	0.4	0.2	0.8	**0.017**
Third	1.2	0.5	2.9	0.611	1.0	0.4	2.1	0.939	0.7	0.3	1.5	0.327
Forth or more	1.3	0.5	3.3	0.641	0.6	0.2	1.4	0.215	0.5	0.2	1.3	0.143
Burnout: Emocional Exhaustion	2.7	1.7	4.1	**< 0.000**	2.5	1.7	3.7	**0.000**	2.6	1.8	4.0	**< 0.000**
Burnout: Depersonalization	1.0	0.8	1.3	0.880	1.0	0.8	1.3	0.877	0.9	0.7	1.1	0.309
Burnout: Personal Accomplishment	0.9	0.6	1.3	0.429	1.3	0.9	1.9	0.168	0.8	0.6	1.2	0.351
WOQOL: Physical domain	0.3	0.2	0.6	**< 0.000**	0.3	0.2	0.6	**0.001**	0.6	0.3	1.2	0.163
WOQOL: Psycological domain	0.1	0.1	0.3	**< 0.000**	0.4	0.2	0.7	**0.003**	0.2	0.1	0.4	**< 0.000**
WOQOL: Relationship	1.0	0.6	1.5	0.876	1.0	0.7	1.4	0.896	0.9	0.6	1.4	0.628
WOQOL: Environment	1.9	1.0	3.6	0.056	1.0	0.6	1.8	0.919	1.3	0.7	2.5	0.338
WOQOL: Quality of life perception	0.8	0.5	1.2	0.235	1.2	0.8	1.8	0.303	1.1	0.7	1.6	0.769
WOQOL: Health satisfaction	1.2	0.8	1.8	0.351	1.0	0.7	1.4	0.823	0.7	0.5	1.0	0.070

*OR* odds ratio, confidence interval, *ref*. reference category

A negative correlation was detected between depression and anxiety symptoms and WHOQOL-BREF physical domain (OR = 0.3, CI = 0.2–0.6, p value = < 0.000; OR = 0.3, CI = 0.2–0.6, *p* = 0.001) and WHOQOL-BREF psychological domain (OR = 0.1, CI = 0.1–0.3p value < 0.000; OR = 0.4, CI = 0.2–.07, *p* = 0.003) and between stress symptoms and WHOQOL-BREF psychological domain (OR = 0.2, CI = 0.1–0.4, p value = < 0.000).

The models did not show correlation between mental health symptoms and demographic information, such as age, relationship status and place of graduation. However, anxiety was positively associated with non clinical, non surgery specialties (OR = 3.9, CI = 1.1–13.8, *p* = 0.034).

## Discussion

This study reported the prevalence of mental health symptoms and its correlates with Burnout Syndrome and quality of life among medical residents of the largest Brazilian academic medical residence service. Our study included a diversified sample, with participants from all medical specialties and in different training years. We had a response rate in absolute number higher than any other Brazilian study in the field [11–14].

Our study showed high rates of emotional exhaustion and depersonalization as confirmed by present literature [2, 3, 11, 12]. The prevalence of depression, anxiety and stress symptoms is also similar to other studies [1–3, 5].

The positive correlation between depression, anxiety symptoms and Burnout Syndrome had already been reported [4, 11, 12]. However, the correlation with stress is less frequently reported. The use of DASS-SF made it possible to perform a broad correlation between Burnout Syndrome and mental health symptoms.

Our findings corroborate the presumption that there is a high rate of mental health impairment and Burnout Syndrome among medical residents/fellows, which should be assessed in different populations in order to guide future resolutions. There are many studies about interventions to prevent and reduce burnout among physicians. Interventions can be either individual- centered, such as mindfulness programs, stress management programs and small group discussions, or structural interventions, for example duty hour limitation policies [19].

The WHOQOL-BREF survey demonstrated average punctuation in all domains and a low overall quality of life satisfaction. Physicians’ quality of life is a complex and multifaceted concept, as individual, professional and organizational factors might affect their capacity to feel well. Growing evidence points to important negative consequences of physicians illnesses to health-care systems, quality of care and patient safety [20].

### Limitations

Our study presents a number of limitations. First of all, it is a cross-sectional study. Although this type of study may be useful to estimate the prevalence of a subject of interest, it does not establish a temporal relationship between events and can present bias. Therefore, a longitudinal study is needed to provide more insight into mental health changes over the course of the year and during medical journey.

Secondly, although we had a satisfactory number of participants in the study, which resulted in an adequate sample size for multivariate modeling, our response rate was 36.8% of those who received an invitation to participate in the survey. This percentage may represent a biased sample, as it may seem likely that those residents/fellows who may present symptoms will show greater interest to answer the survey. In order to increase the response rate and improve the heterogeneity of the sample, a multicenter study should be considered.

In order to perform statistical analyses, it was necessary to categorize the DASS-SF and MBI scores. This strategy may result in loss of information. Another limitation is the absence of information on gender, as we decided not to require in order to reduce institutional exposure.

## Conclusion

In conclusion, medical residents and fellow physicians of the largest Brazilian academic health system present a concerning prevalence of mental health symptoms, which are positively correlated with Burnout Syndrome. Furthermore, overall quality of life satisfaction is low. Multicenter studies with longitudinal design are needed in order to confirm the hypotheses formulated in this study. Studies in this field are important to promote debate about medical residence and to guide future interventions to improve mental health among doctors in training.

## Data Availability

The datasets used and/or analyses during the current study are available from the corresponding author if requested.

## Abbreviations

DASS-SF: Depression anxiety and stress scale-short gorm
MBI: Maslach burnout inventory
OR: Odds Ratio
REDCap: Research electronic data capture
WHOQOL-BREF: World Health organization quality of life-bref assessment instrument
